# Immunological Studies to Understand Hybrid/Recombinant Variants of SARS-CoV-2

**DOI:** 10.3390/vaccines11010045

**Published:** 2022-12-25

**Authors:** Vivek P. Chavda, Toshika Mishra, Suneetha Vuppu

**Affiliations:** 1 Department of Pharmaceutics and Pharmaceutical Technology, LM College of Pharmacy, Ahmedabad 380009, Gujarat, India; 2 Department of Biotechnology, Science, Innovation, and Society Research Lab. 115, Hexagon (SMV), Vellore Institute of Technology, Vellore 632014, Tamil Nadu, India

**Keywords:** COVID-19, SARS-CoV-2, hybrid, variants of concern, immunological assays

## Abstract

The zoonotic SARS-CoV-2 virus was present before the onset of the pandemic. It undergoes evolution, adaptation, and selection to develop variants that gain high transmission rates and virulence, resulting in the pandemic. Structurally, the spike protein of the virus is required for binding to ACE2 receptors of the host cells. The gene coding for the spike is known to have a high propensity of mutations, as a result generating numerous variants. The variants can be generated by random point mutations or recombination during replication. However, SARS-CoV-2 can also produce hybrid variants on co-infection of the host by two distinct lineages of the virus. The genomic sequences of the two variants undergo recombination to produce the hybrid variants. Additionally, these sub-variants also contain numerous mutations from both the parent variants, as well as some novel mutations unique to the hybrids. The hybrid variants (XD, XE, and XF) can be identified through numerous techniques, such as peak PCR, NAAT, and hybrid capture SARS-CoV-2 NGS (next generation sequencing) assay, etc., but the most accurate approach is genome sequencing. There are numerous immunological diagnostic assays, such as ELISA, chemiluminescence immunoassay, flow-cytometry-based approaches, electrochemiluminescence immunoassays, neutralization assays, etc., that are also designed and developed to provide an understanding of the hybrid variants, their pathogenesis, and other reactions. The objective of our study is to comprehensively analyze the variants of SARS-CoV-2, especially the hybrid variants. We have also discussed the techniques available for the identification of hybrids, as well as the immunological assays and studies for analyzing the hybrid variants.

## 1. Introduction

The novel coronavirus that caused the coronavirus (COVID-19) pandemic, Severe Acute Respiratory Syndrome Coronavirus 2 (SARS-CoV-2), belongs to a family of viruses known for causing rare but serious infections, particularly critical respiratory conditions that result in greater mortality and morbidity [1]. SARS-CoV-2 or HCoV-19 is the seventh coronavirus that leads to infections in humans. SARS-CoV, Middle East Respiratory Syndrome (MERS-CoV), and SARS-CoV-2 are the viral strains that result in severe and complex infection, whereas the strains HKU1, NL63, OC43, and 229E cause minor symptoms [2]. The origin of any organism is determined by analyzing its genetic makeup to track the course of evolution. This approach is also applied to the case of novel coronavirus to identify its source of origin. Studies reveal that SARS-CoV-2 has a zoonotic origin, as it was found that this strain of virus exhibits similarity with coronaviruses isolated from bats, specifically horseshoe bats (*Rhinolophus*) in China and pangolins [1,3]. It is hypothesized that SARS-CoV-2 was derived from the Sarbecovirus of the Betacoronavirus genus in the horseshoe bat which transferred it to the animal host, the civet cat [3]. There are several signatures present in SARS-CoV-2 indicating prior zoonotic events [1].

Studies have revealed that SARS-CoV-2 originated numerous years well before the outbreak of the pandemic but could not trigger an epidemic or pandemic [4,5]. This ability is gained by following Charles Darwin’s model of evolution, adaptation, and selection. Viruses gain this environment to evolve in the living host, further acquiring the ability to infect numerous individuals of the population by enhanced virulence and transmission rates. This is evident in the instance of SARS-CoV-2 [4].

These viruses exhibit a strong affinity to human Angiotensin Converting Enzyme 2 (ACE2) receptors. Studies have revealed that the SARS-CoV-2 virus acquired this high affinity for human ACE2 receptors 7–50 years ago. This raises a concern that numerous animal viruses that have gained affinity toward human receptors through a similar course may randomly evolve into a virulent strain that can lead to epidemic and pandemic situations. Furthermore, the future adaptations of SARS-CoV-2 are still a great cause for concern, as there is a lack of tools and techniques to predict the viruses’ future course of evolution [5].

The World Health Organization (WHO) on 23 December 2022 reported 651,918,402 confirmed cases of COVID-19 with 6,656,601 deaths [6]. The epidemiology of the infection caused by the virus has grown considerably over time due to approaches and steps taken to prevent or control the transmission, as well as the design and development of efficient treatment options to decrease the magnitude of the infection and fatalities. Various initiatives, such as the vaccination campaign, have significantly reduced the magnitude and mortality rates of infection in areas with high vaccination rates [7,8].

## 2. Variants of SARS-CoV-2

SARS-CoV-2 is a prevalent RNA virus that evolves and adapts to human hosts through mutations. The replication of SARS-CoV-2 is performed with the help of RNA polymerase with inadequate fidelity, which results in the formation of mutations in the nucleic acid [9]. This results in the development of numerous novel variants with characteristics that differ from the parent strain. One amino acid change can give the virus the potential to elude immune response, complicating the development of effective vaccines. Although these occurrences are a rare phenomenon, the development of such mutations confers alterations in pathogenicity, infectivity, transmissibility, and/or antigenicity [10]. Mutations can also make the virus unresponsive to the available treatment approaches [11]. The spike (S) glycoprotein is essential for infection and a key target for neutralizing antibody recognition. The mutations in the S gene lead to “viral fitness” thus, developing variants of concern favor their survival by enhancing properties, such as affinity to the ACE-2 receptor, pathogenicity, multiplication, increased transmission, resistance to antibodies, and immune evasion [12]. Studies have revealed that mutations in the receptor-binding domain in the region of the K417-E484-N501 triad causes the virus to evade the immune response by inhibiting the binding to class 1 and 2 antibodies. Additionally, these mutations also enable the evasion of immunity by vaccination and infection as well as prophylactic therapies [9].

The changes in the genetic code of the virus happen either by random point mutations (Alpha, Beta, Gamma, Mu, Delta, and Omicron) or recombination (coronaviruses including novel hybrid variants, influenza virus, HIV, etc.,) during viral replication. The variants are classified into lineages based on their evolution from a common ancestor [13,14]. The investigations on the viral genomes focus on tracking the global transmission of the virus, analyzing the local outbreaks, and promoting public health policies. This is facilitated by the generation and sharing of viral genomic sequences [10]. The Centers for Disease Control and Prevention (CDC) reported that the SARS-CoV-2 Interagency Group (SIG) constituted by the US government classified the virus variants into four broad classes: Variants of Concern (VOCs), Variants Being Monitored (VBMs), Variants of Interest (VOIs), and Variants of High Consequence (VOHCs). The classification is not rigid and the variants are continuously monitored to reclassify them depending on alterations in their attributes and prevalence.

The first major variant of concern was Alpha (B.1.1.7), identified in the U.K. [11]. A study recognized five important Variants of Concern that had a widespread impact on various parts of the world. This includes lineages B.1.1.7, B.1.351, P.1, B.1.617.2, and B.1.1.529 which were first detected in the U.K., South Africa, Brazil, India, and multiple countries, respectively [12]. The outbreak of infections by variant B.1.1.7 illustrated the significance of VOCs. Until that point, there existed a lack of sufficient proof for the fact that modifications to the RNA content of SARS-CoV-2 significantly improved viral fitness [15]. Table 1 shows the details of some important Variants of Concern (VOCs) that had a global impact.

The scientific community follows an established nomenclature system provided by the Global Initiative on Sharing All Influenza Data (GISAID), NextStrain, and Pango for the naming and tracking of the lineages of the SARS-CoV-2 virus. The WHO has reported that the currently circulating VOC is Omicron variant B.1.1.529, as provided by the data from the Pango lineage [19,20]. The variants of Omicron BA.1, BA.2, BA.3, BA.4, BA.5, and descendent lineages are being monitored by the WHO, as they are suspected to become a threat to human health if not monitored efficiently [20]. The Technical Advisory Group on SARS-CoV-2 Virus Evolution (TAG-VE) declared Delta (B.1.617.2) and Omicron (B.1.1.529) as currently circulating VOCs because of their greater virulence and transmissibility [21]. The Omicron variant has alarmingly high transmission rates than other VOCs; however, several studies have reported that the variant exhibits lower pathogenicity than other VOCs [22]. The mutations of Omicron make it antigenically distinct from the parental viruses and other prevalent VOCs, resulting in a reduction in antibody neutralization on vaccination or natural infection [23].

## 3. Hybrid Variants

Hybrid variants are a growing cause of concern in the current scenario because they have the potential to again increase positive cases globally by gaining enhanced virulence and transmission rates on recombination between two variants of distinct lineages [14].

The variants of SAR-CoV-2 are usually generated by point mutation; however, the hybrid variants are generated by recombination between the genetic content of two different variants or lineages. The cause of mutations is attributed to the erroneous proofreading activity of RNA polymerase. Studies prove that the mechanism to produce novel variants of SARS-CoV-2 by point mutation is of lower efficiency when compared with recombination. Nonetheless, the initial high prevalence of SARS-CoV-2 and COVID-19 cases globally aided the virus in producing successful variants through point mutations. Furthermore, the occurrence of several peaks of COVID-19 incidences indicates the characteristics of the virus-like high transmission rates and retention of survival benefits facilitated by the accumulation of missense mutations. The recombination is the advantageous mechanism for SARS-CoV-2 as it happens between two different lineages with enhanced features, such as higher virulence and transmission rates, leading to continuous circulation and evolution of the virus [24]. The recombinations are classified based on the site of the crossover. In the case of homologous recombination, the crossover happens at the same location in both parental strands whereas, in non-homologous recombination, the crossover takes place at different locations giving rise to anomalous structures. Recombination in RNA viruses is regarded as a result of the selection of genomic traits for the regulation of gene expression, as well as the natural selection of certain genotypes produced by this approach [25].

Studies reveal that during the present pandemic situation, there are numerous variants of SARS-CoV-2 that are co-existing in the environment. In cases where two variants co-infect a host, they undergo recombination of the genetic material in the host cell, generating a novel hybrid subvariant, such as Deltacron (XD and XF) and XE. The Deltacron subvariants, as the name suggests, originate from Delta and Omicron lineages [21]. It was first reported in France [26]. The hybrid subvariants, Deltacron and XE, had rapidly transmitted to all six continents. The nomenclature of hybrid variants contains “X” to signify the recombination between two different lineages of the virus [21]. The XD, XE, and XF are the hybrid subvariants generated from the Omicron variant by recombination. XD and XF are hybrids created from lineages of Delta and Omicron strains. According to the formal nomenclature, Deltacron is denoted as BA.1xAY.4. Due to its high potential for future outbreaks, Deltacron is categorized in the list of Variants being Monitored (VBMs) [21]. XD was created by recombination between the Delta and BA.1 variant of Omicron, whereas recombination of BA.1 and UK Delta variants created the XF variant [14,24]. On the other hand, the XE subvariant was created by recombination between BA.1 and BA.2 variants of Omicron [27]. The unique mutation found in the XD hybrid is at the site NSP2 E172D. Studies report that the XD subvariants infect all age groups and genders. The spike proteins, as well as the structural proteins of XE, are obtained from the BA.2 variant of Omicron where the 5′ region of the genome is from BA.1. Several studies have reported that the hybrids with either the spike or structural protein from a single parental lineage exhibit similarity to its corresponding parent. Mutations at the site NSP3 C3241T, V1069I, and NSP12 C14599T are the three important characteristic mutations identified in XE but absent in BA.1 and BA.2 [26]. The subvariant XE contains the mutations corresponding to BA.1 for the non-structural protein (NSP) region 1–6, whereas the remaining regions of the genome contain the mutations corresponding to BA.2. Furthermore, there are also three exclusive mutations (C3241T at the NSP3 region, C14599T at the NSP12 region, and V1069I at the NSP3 region) in XE that is absent in the BA.1 and BA.2 genome. The mutation at V1069I leads to the cleavage of viral proteins [28]. It possesses 10-fold greater transmission rates than the parent Omicron variant [27]. The XE subvariant gained high global prevalence, especially in the UK, Japan, China, and Canada. Consequently, this subvariant is being tracked and monitored by the WHO and included in the list of VOCs [21,27]. Studies have also reported the BNT162b2 vaccines are ineffective in eliciting an immune response in patients infected by XE subvariant [21]. Further, the WHO emphasized the systematic tracking of XE predicting its high transmissibility compared to any other strain or variant of SARS-CoV-2 [26]. There are several other variants, such as XQ, XG, XJ, and XK discovered in European countries, including the UK, Denmark, Finland, and Belgium, respectively [21]. A recent study has reported that the Omicron variant has also emerged due to recombination between the parent strain of SARS-CoV-2 with the B.35 lineage [26].

Recombination is typically used to create hybrid variants; however, certain genome alterations have also been discovered to be associated with improved traits of the variant, including resistance to therapy, evasion of immunity provided by infection or vaccination, increased pathogenicity, etc. [9]. The emergence of novel hybrids is attributed to the overall low vaccination rate globally. This allows the virus to survive and circulate in individuals that belong to unvaccinated children and adult populations, immunosuppressed patients, or adult populations over 75 years of age. The easing of COVID-19 countermeasures and curbs also provides an environment to circulate and develop recombinants with other co-existing variants [27]. Recombination can generate highly virulent variants that result in enhanced fitness of the variant for survival in the environment. A significant raise in vaccination, especially for the susceptible population, is essentially required to combat the infections by the hybrid variants [21]. When compared with the other variants of SARS-CoV-2, the hybrid variants of the virus exhibit high transmission rates and global circulation, as is evident from their prevalence on the five continents of the world [24].

Studies report that there are over 1000 recombinations that are possible to generate hybrid variants, some of which may possess increased pathogenicity compared to the ancestral lineages. In the case of sub-lineage XL (hybrids with recombinations between the BA.1 and BA.2 variants of Omicron), the recombination is detected by identifying unique mutations in an ORF1a gene at the Leu204Phe, Val1887Ile, and Ser22981syn sites. Recombinant genomes are found to be associated with notable challenges in bioinformatics-based approaches for the detection of theiants in clinical samples [29].

## 4. Techniques for Identification of Hybrid Variant

**Figure 1:**
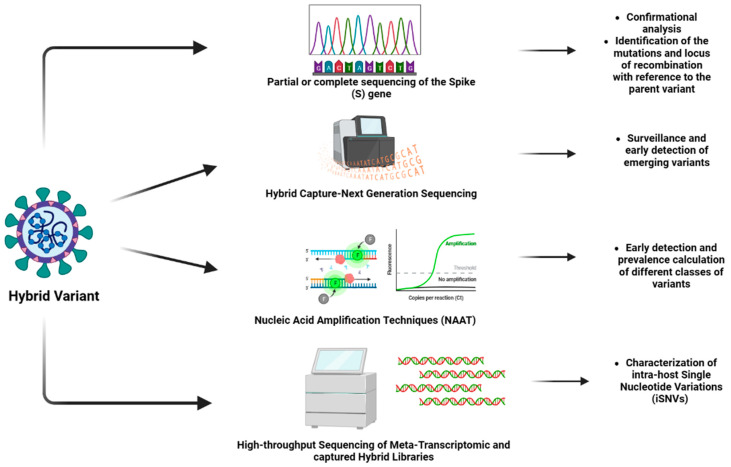
Different techniques for the identification of hybrid variants (Created by BioRender.com).

The hybrids of SARS-CoV-2 are widely studied and monitored to understand their evolution as well as tracked to prevent the outbreak of any variant of high transmissibility and virulence. There are several techniques employed to identify hybrid variants. With the advancement in technology, techniques that yield more accurate results are being developed globally. Figure 1 shows different techniques for the identification of hybrid variants.

Many companies and institutes involved in designing novel diagnostic techniques have developed combination approaches that combine two or three different techniques for more precise detection. Most of the approaches have focused on using RT-PCR in combination with techniques such as sequencing, analysis of melting curves, qPCR, etc., for hybrid determination. This includes methods such as the SARS-CoV-2 variant assay involving RT-PCR and sequencing, the multitarget designed assay comprising of RT-PCR and analysis of the melting curve, and the multivariant deletion assay involving RT-PCR and qPCR, etc. [30].

The most widely used approach for this purpose is genomic sequencing (which can be whole genome sequencing or partial or complete sequencing of the spike (S) gene of the virus. The sequencing technique is the confirmation analysis for the identification of the variants [31]. With a large dataset of sequences of SARS-CoV-2 variants available to analyze the sequence of SARS-CoV-2, this technique is efficient in identifying the mutations and locus of recombination concerning the parent variant. The technique can be used for designing diagnostic approaches, as it is efficient in identifying the conserved domains and performing phylogenetic analysis [30].

A study focused on developing a novel method validated as a “hybrid capture SARS-CoV-2 NGS (Next Generation Sequencing) assay”, designed by a combination of double-stranded DNA biotin-labeled probes and complimentary software. This approach has received emergency-use authorization from the FDA and is found to be greatly useful in the surveillance and early detection of emerging variants, thus aiming at the prevention and mitigation of future outbreaks [32]. In another study, a fast diagnostic technique was designed using the portable instrument *peak*PCR. The assay is designed to detect two single nucleotide polymorphisms (E484K and N501Y), as well as the deletion of the spike gene (HV69/70). This technique provides better results in testing the isolated RNA samples from the VOCs [33].

An assay based on nucleic acid amplification techniques (NAATs) is another diagnostic screening approach that can be utilized to identify the hybrid variants. This facilitates early detection and prevalence calculation of different classes of variants, including VOCs, VOIs, and VBMs. It is preferred to perform sequencing for confirmation in NAAT-based diagnostic screening. The sequencing data obtained should be deposited in suitable databases, such as the GISAID, and other public databases to share the data on novel variants with the scientific community [31].

Numerous commercial rapid assays have been designed for identifying the variants, such as the SARS-CoV-2 variant direct assay, that detects the mutations in the spike protein at the N501Y, E484K, E484Q, and L452R sites. In this method, the samples from the nasopharyngeal and nasal swabs can be used directly, without the requirement for the extraction of RNA [30].

There is another approach utilized for the identification and analysis of hybrid variants, which involves high-throughput sequencing of meta-transcriptomic and captured hybrid libraries. The approach involves the characterization of intra-host single nucleotide variations (iSNVs) which frequently occurs in individuals infected with SARS-CoV-2 [34]. Table 2 summarizes the important techniques used for the identification of the hybrid variants. The advancement in technology has drastically improved the field of pharmacology. The diagnosis of conditions is becoming relatively easier and more accurate. The design and development of efficient therapeutics, as well as vaccines for treatment and prevention, respectively, also require less time in the current situation. Additionally, these approaches are also aiming to become cost-effective so that individuals from all economic strata can access them.

## 5. Immunological Studies and Assays

The frequent emergence of novel variants of the virus is a serious concern and, as a consequence, it becomes essential to update the diagnostic tests to increase the range of detection even to the new variants. Three parameters should be ideally maintained in the diagnostic assays, which include sensitivity, specificity, and accuracy. The immunological assays are designed to shed light on the efficacy of the host in combating viral infection and to some extent provide insight into the pathobiology of the virus [16].

In the current scenario, serological or immunological assays are being performed as additional tests to enhance the sensitivity of the diagnostic tests. These approaches help to understand the mechanism of immune evasion by the variants of SARS-CoV-2, including the hybrid variants [44]. In this section, we discuss all the potential immunological assays that can be utilized for the accurate detection and understanding of hybrid variants. To date, there has been no approach or technique designed specifically for the hybrid variants. Thus, the proposed approaches can be utilized for the diagnosis of the hybrid variants with modifications in the assay parameters (for example, using antigens specific to hybrid variants) for obtaining accurate results.

**Figure 2:**
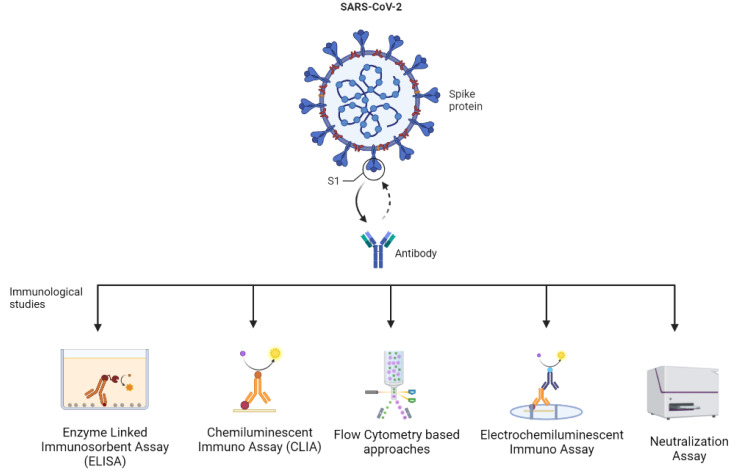
Different immunological assays to study hybrid variants (Created by BioRender.com).

The tests or assays provide data on antibody titer, especially that of neutralizing antibodies, determine the magnitude and time of immunity obtained on infection by SARS-CoV-2, predict the risk of becoming re-infected, and the requirement of a booster dose. The immunological studies are based on technologies such as immunoassays (for example, the enzyme-linked immunosorbent assay—ELISA), chemiluminescent immunoassays (CLIA), electrochemiluminescence immunoassays, neutralization assays, techniques based on flow cytometry, etc. (Figure 2) [16,34]. In addition to these, new technologies with benefits, such as being easy-to-use and having a high-speed detection, are developed. This includes assays, such as point-of-care lateral flow immunochromatographic assays, which have gained unprecedented popularity at the current time [34].

The variant-specific antibodies serve as a reliable biomarker of the previous infection by the virus as well as the stage of infection. A study discovered that 40% of positive cases reported measurable levels of IgM and IgG during the first 7 days of infection, whereas cross-antibody levels are observed after 15 days. The ELISA test can be used for the rapid detection and quantification of IgA, IgM, and IgG against the receptor-binding domain of the variants of SARS-CoV-2. These receptor-binding domains are the target sites for neutralizing antibodies [45]. This approach is found to have high sensitivity but low specificity [46]. Similar to ELISA, CLIA is also based upon the binding between the antigen of the viral variants and specific antibodies of the host. However, it utilizes a chemical probe to exhibit a positive reaction. This approach is reported to be rapid with high accuracy, as well as sensitivity and specificity. Studies have revealed that the technique of lateral flow immunoassay (LFIA) has high potential in the diagnosis of COVID-19, targeting the spike protein of the virus. The spike protein-specific IgG is a with a fluorescent compound to detect the positive reaction. It is a simple, cost-effective, rapid, and easy-to-perform test [47]. In the case of the electro-chemiluminescent assay, the viral antigens, including the spike protein, RBD, and coat proteins, are used to detect the specific IgG with high sensitivity and specificity [48].

It has been observed that the new emerging variants and subvariants possess the ability to evade the immune response by escaping neutralizing antibodies. The neutralizing antibodies are generated in a host on vaccination or past incidence of infection. They have the ability to inhibit the interaction between the viral spike protein and the host Angiotensin Converting Enzyme 2 (ACE-2) receptor. The activity of neutralizing antibodies can be estimated to detect a person’s current status of protection against specific variants of SARS-CoV-2. These neutralization assays employ techniques, such as live virus plaque reduction and ACE-2 binding inhibition assays, to study the impact of neutralizing antibodies on hybrid variants [49]. The alterations in the genetic material are predicted to facilitate the evasion of neutralization. Techniques such as the plaque reduction assay can also be utilized to identify effective vaccines for particular hybrid variants. The titer of neutralizing antibodies is reported to be different for different variants of the virus [44]. Recently, another innovative neutralization assay involving the binding of the spike protein of the virus with the host ACE-2 receptor is developed. It is a quick, easy, and sensitive approach that involves high-throughput screening of therapeutic compounds such as antibodies, small molecules, and peptides. This technique can be used for the validation of novel vaccine formulations, potential therapeutics, and screening of inhibitors [50]. The activity of neutralizing antibodies can be detected by utilizing a novel high-throughput approach using a multiplexed flow cytometer-based assay. This approach is applicable to multiple variants with different antigens and so it can be used to distinguish between different variants and subvariants of SARS-CoV-2 [49]. With the advancement in technology, many of the discussed assays are performed in a multiplexed setting that enables the testing of multiple samples simultaneously.

ELISA, CLIA, electro-chemiluminescent assays, and flow cytometer-based assays can potentially be used as sensitive and specific diagnostic assays for hybrid variants, whereas neutralization assays may be used to understand the pathogenesis and identify prospective therapeutics and vaccines for hybrid variants. Thus, the immunological assays can not only be utilized for accurate diagnosis of the hybrid variants but also propose effective and specific prophylactic approaches (therapeutics) and preventive measures (vaccinations). These approaches will potentially be accepted and performed in the near future. The advantages and limitations of the discussed methods are mentioned in Table 3.

There are numerous novel assays submitted to the FDA for emergency-use authorization; however, studies reveal that they lack standardization and inaccurately detect the binding antibodies instead of antigen-specific antibodies [60]. Another limitation in designing an efficient immunological assay is the variability of the spike protein due to its high propensity to mutate which complicates the standardization of immunological assays and vaccines [61]. A recent study reported that the spike protein of the Omicron variant has about 30 unparalleled mutations, conferring it the property of immune evasion and increasing the risk of re-infection. The study examined the measures of cross-reaction of antibodies before and after vaccination against specific SARS-CoV-2 spike and receptor-binding domain (RBD) proteins in 48 patients who were recovering from COVID-19 infection. The results reveal an increase in Alpha and Delta RBD antibodies, and a reduced reaction to Beta and Omicron RBD antibodies. Following the structural investigation, it was discovered that the Beta and Omicron RBD shared an immune escape mechanism consisting of residues that are manipulated by these VOCs [9]. Thus, this approach can be utilized to distinguish the hybrid variants by analyzing the cross-reactive antibodies.

## 6. Challenges in the Immunological Analysis of Hybrid Variants

There are several challenges associated with the immunological assays for hybrid variants. Although the neutralization assays are recognized as the gold standard for the analysis of neutralizing antibodies, the assays require exclusive facilities with biosafety level 3 certification. Neutralization assays in addition to ELISA tests essentially require skilled technicians to perform the tests. Another major limitation of immunological assays is that different immunoglobins are detected at different time intervals in the host. IgM is detected at an average of 5 days post-onset of symptoms, whereas IgG is detected after about 14 days. A major concern associated with immunological or serological assays is the production of false negative outputs due to the cross-reactivity of antibodies [62]. This cross-reactivity is attributed to the predominance of several hybrid variants of SARS-CoV-2 that have variations in the spike and nucleocapsid proteins, which are the major antigens used for the diagnosis of specific variants [63]. The cross-reactions lead to low specificity of the assay, providing inaccurate results even in cases of low percentages of false-positive results. The recombination between genomes of two distinct variants or lineages that leads to the development of hybrid variants results in the appearance of exchangeable neutralization epitopes. Consequently, these epitopes evade the immune response and infect protected populations [64]. These variations in epitopes also reduce the specificity of the immunological assays, giving rise to the need to development of assays specific to each variant.

## 7. Conclusions and Future Prospects

COVID-19 is a complex infection majorly attributed to the high propensity of mutations in its spike proteins that confer alterations in the attributes and transmission rates, as well as virulence. The virus undergoes frequent random point mutations or recombination during replication which results in the development of new variants. Further, two distinct lineages of SARS-CoV-2 can also recombine to form hybrid variants that can be monitored to track conversion into a variant of concern. There are numerous methods to identify the hybrid variants; however, the most accurate approach is genome sequencing. Numerous immunological diagnostic tests are also designed to identify and examine the variants of SARS-CoV-2.

Mutations and recombinations in the viral genome can render the currently available drugs and vaccines ineffective. Therefore, the understanding of various aspects of hybrid variants such as the mechanism of development, mutations involved, pathogenicity, transmission rate, resistance to therapies and vaccines, etc., is essential to design more efficient drugs and vaccines for the control of the infection. The hybrid variants require to be constantly monitored and comprehensively analyzed, as they can become converted to a variant of concern. The invention of novel techniques to detect or diagnose the variants, in addition to immunological studies and assays to understand them, are the need of the hour to control the transmission of these variants. These approaches further facilitate improvement in our understanding of viral mechanisms and evolution, which can benefit easy mitigation and control of future outbreaks of viral infections. The immunological assays described have the potential to be used for efficient and accurate diagnosis of the hybrid variants in the near future.

## Figures and Tables

**Table 1:** Variants of Concern (VOCs) with a global impact (prior to the origin of Omicron).

Sl. No.	Variants of Concern (VOCs)	Mutations	Property of the Variant	References
1.	Alpha (B.1.1.7)	A total of 23 mutations (17 amino acid changes) from the first strain that was discovered in Wuhan. Notable mutations: spike D614G, spike N501Y, and spike HV-69–70 deletions.	Increased transmissibility and infectivity. About 70% greater transmission rate than the Wuhan-1-strain.	[16]
2.	Beta (B.1.351)	Notable mutations: multiple mutations in the S protein, three in the RBD (N501Y, E484K, and K417N).	Increased transmissibility. Reduced elimination by monoclonal antibodies, convalescent plasma, and post-vaccination sera.	[16]
3.	Gamma (P1)	Seventeen mutations (eleven amino acid changes). Notable mutations: N501Y, E484K, and K417T.	Increased risk of transmission. Dampened neutralizing humoral immunity.	[16]
4.	Delta (B.1.617.2)	Notable mutations: 12 mutations, 10 of which in the S-protein (T19R, G142D, 156del, 157del, R158G, L452R, T478K, D614G, P681R, and D950N). Contains major mutations in RBD and NBD-containing S1 subunits.	Most transmissible. A total of 60% more contagious than alpha. Patients face longer periods of infection. Increased immune evasion and infectivity. Interference with the host antibody response.	[16,17]
5.	Omicron (B.1.1.529)	The spike protein contains 32 amino acid mutations. Notable mutations: K417N, E484K, N501Y, D614G, and T478K.	A total of 5–11 times greater mutations in spike proteins than in other variants. Increased transmission rates and evasion of an immune response.	[18,19]

**Table 2:** Techniques for the identification of hybrid variants.

Technique	Description	Comments	Output	Advantages	Limitations	Reference
Whole genome sequencing or partial or complete sequencing of the spike protein	The nucleic acid samples are fragmented into smaller segments. These sequences are independently decoded followed by the alignment of all the sequences using computer algorithms.	This technique is facilitated by high-throughput sequencing approaches. The sequencer identifies the nucleotide bases that make up the sequence of the nucleic acid chain. Computer-based tools are used for comparison as well as identification of the variations.	The result obtained is nucleotide sequence on a computer system.	Confirmational analysis. Rapid identification. Facilitate the development of novel diagnostic techniques. Useful in surveillance of the hybrid variants, their transmission, activity, evolution, and generation of new variants.	Expensive Requires technical support	[35,36,37,38]
Hybrid capture SARS-CoV-2 NGS (Next Generation Sequencing) assay	It utilizes double-stranded probes that are labeled with biotin for the purpose of panel design integrated with software for the detection and mapping of the hybrid variants. Additionally, the microbiomes in the nasopharyngeal tract.	First, the assay has to be authorized by the FDA for emergency use. This technique is useful for improved monitoring and early diagnosis of infection by hybrid variants.	The result obtained is nucleotide sequence on a computer system.	Applicable in identification of other pathogens like the influenza virus. Facilitates the understanding of dynamics and the evolutionary profile of pathogens (hybrid variant). The approach is exclusive, sensitive, and specific.	Labor-intensive Requires greater quantities of input for the process	[39]
Nucleic acid amplification techniques (NAAT)	The procedure is a sensitive diagnostic test that is based on the amplification of the viral genome which facilitates the detection of RNA of the virus.	This technique is a rapid, industry-standard approach. It basically involves real-time polymerase chain reactions (RT-PCR), CRISPR- related amplification, loop-mediated isothermal amplification, strand displacement amplification, as well as a ligase chain reaction.	The result of the amplification is studied using fluorescent probes computer system linked to the PCR machine.	Sensitive. Specific.	Expensive Complex procedure Time-consuming method	[40,41,42]
SNP Assays	The technique enables rapid estimation of prevalent variants with specific mutations by RT-PCR assays that target single polymorphism.	The effectiveness of the technique can be enhanced by integrating it with whole genome sequencing. The melting curve analysis of RT-PCR has been used commercially for the detection of Variants of Concern.	The result of the amplification is studied using fluorescent probes computer system linked to the PCR machine.	Modified qualitative RT-PCR can be used for the detection of different Omicron lineage.	Inefficient in the identification of novel variants that lack specific SNP Numerous commercially available assays are ineffective for the detection of Omicron	[31]
Reverse transcription loop-mediated isothermal amplification	Alternative molecular method for identification of variants of SARS-CoV-2.	This technique when integrated with CRISPR-Cas13 can efficiently detect the hybrid variants of SARS-CoV-2 with 100% specificity, as well as 83% sensitivity.	The result is observed by visual observation of turbidity or fluorescence.	Faster results. High specificity and sensitivity.	Current, cannot differentiate between specific Variants of Concern Require more validation studies	[31,43]
High-throughput sequencing of meta-transcriptomic and captured hybrid libraries	The sequences of the sample are mapped on a pre-determined database with genomes of coronaviridae that are used as references to eliminate low-quality data. Further, intra-host variants are identified.	This technique involves the characterization of intra-host single nucleotide variations (iSNVs). In this approach, both metatranscriptomic and hybrid-capture sequencing are performed to minimize any probability of error during sequencing.	The result obtained is nucleotide sequence on a computer system.	Identify all pathogenic organisms in the sample. Useful in understanding the interaction between the host and pathogen (virus), especially RNA viruses. Efficiently analyze the mutations and determine the course of evolution of the virus into new variants. Vigorous estimation of viral load.	Expensive Labor-intensive Requires technical suppose Complex methodology	[34]

**Table 3:** Potential immunological assays for detection and understanding of hybrid variants.

Technique	Description	Advantages	Limitations	References
Enzyme-Linked Immunosorbent Assay (ELISA)	It facilitates rapid detection and quantification of IgA, IgM, and IgG against coat proteins, spike proteins, and the receptor-binding domain (RBD).	High sensitivity High specificity (in some cases) Detection of IgG is essential for understanding antibody-mediated immunity and reaction to the vaccine Easy and rapid	Low specificity (suggested by some studies) False positive results because of conservation of antigenic regions among various variants of SARS-CoV-2 The RBD-based ELISA is a time-consuming process that can take over 4 h to complete	[45,46,51]
Chemiluminescent Immuno Assay (CLIA)	It is based on the high binding affinity of the antigen of a viral variant with specific antibodies of the host. The technique requires a chemical probe to exhibit a positive reaction.	Raised sensitivity Dynamic range Rapid and accurate with high sensitivity and specificity Decreased incubation period A higher amplitude of the signal	Limited detection of antigen Expensive Use of closed analytical systems.	[47,52]
Electro-Chemiluminescent Assay	The viral antigens are used to detect IgG corresponding to it.	High sensitivity and specificity Low background noise Easy to control the counterparts Rapid Detect multiple biomarkers	Electrode fouling occurs frequently Cross-reaction between indicators of the electro-chemiluminescent assay	[48,53,54,55]
Flow Cytometer-based Approaches	A novel high-throughput approach using a multiplexed flow cytometer-based assay. It can be used for a deep analysis of the immune system of people in different stages of infection.	High sensitivity Rapid Analyze a wide range of antibodies against the spike protein	False positive detection Need proper planning before the progression of the protocol (e.g., preparation of cell culture) The collection of samples is time-consuming	[49,56,57]
Neutralization Assay	It involves techniques such as live virus plaque reduction (titer of neutralizing antibodies) and ACE-2 binding inhibition assays (binding of spike protein to the host ACE-2 receptor. It is used for validation of novel vaccine formulations, potential therapeutics, and screening of inhibitors.	Rapid Easy High sensitivity Performs high-throughput screening The gold standard for analysis of antibody-mediated immunity in vaccinated people or those pre-exposed to the pathogen	Tedious Cannot be used for routinely screening large samples Fast degradation of neutralizing antibodies	[49,50,58,59]

## Data Availability

Not applicable.
